# Social realities in remote villages: Infant and young child feeding in Kirewa, Uganda

**DOI:** 10.1371/journal.pgph.0003016

**Published:** 2024-09-10

**Authors:** Lauriina Schneider, Katja Korhonen, Sari Ollila, Marja Mutanen

**Affiliations:** Department of Food and Nutrition, University of Helsinki, Helsinki, Finland; PLOS: Public Library of Science, UNITED STATES OF AMERICA

## Abstract

Understanding infant and young child feeding (IYCF) practices in Africa requires an examination of the social context. Social relationships influence people through mechanisms such as social support, social influence, social engagement, access to resources and negative social interactions. This study explores how these mechanisms manifest in IYCF in remote villages in Uganda. In 2018, we conducted two focus group discussions each with mothers, fathers and grandparents, ande interviews with two clan leaders, six village health teamers (VHT) and four healthcare workers (HCW). We deductively searched the data for any indications of elements that could influence child feeding and health using the psychosocial mechanisms of social support, social influence, social engagement, access to resources and negative social interactions as the broader themes. The manifestation of social support involved practical help from mothers-in-law (MIL), financial contributions from fathers, and informational, instrumental, emotional and appraisal support from VHTs. Social influence by MILs mainly concerned the transmission of food-related beliefs and pressure to have many children. The social engagement of young mothers was restricted. Access to resources was stratified and affected by poverty, patriarchy, and knowledge of HCWs and VHTs. Negative social interactions included physical abuse, alcoholism, and fear-based relationships. We found the different psychosocial mechanisms to construct a useful framework for describing the social reality surrounding IYCF. Changing attitudes towards family planning, involving fathers in IYCF, and strengthening the position of VHTs as family advisers can potentially improve IYCF in rural Uganda.

## 1. Introduction

As poverty, conflict and climate change threaten food and nutrition security [1], efforts to improve infant and young child feeding (IYCF) practices have mainly been facilitated through education, food provision and supplementation interventions [2–4]. Although the percentage of stunted children is slowly declining, the number of stunted under-5-year-olds in Sub-Saharan Africa has as a result of rapid population growth increased from 50.4 million in 2000 to 59.4 million in 2022 [5]. Reviews have indicated that education, socioeconomic status, maternal nutrition, hygiene, healthcare services and family planning affect child growth faltering [6, 7].

Understanding improper IYCF practices in Africa requires an additional examination of the social context wherein IYCF occurs, as these are social practices that are affected by the surrounding cultural context and involve multiple persons in a mother’s social network [8]. For example, the role of grandmothers as cultural advisers has been recorded to influence IYCF practices [9], while fathers are usually seen as providers uninvolved in childcare [10, 11]. In addition, the cultural context of IYCF includes many cultural beliefs such as the inability of mothers to breastfeed while pregnant, cultural inhibitions about certain foods which are nutritious for small children, and beliefs about the ability of small children to eat or digest nutritious foods; such beliefs have been barriers to both exclusive breastfeeding and proper complementary feeding [12–17]. The studies on social networks in Africa mostly focus on contraceptive use [e.g. 18, 19] and HIV [e.g. 20, 21]. However, social networks have impacted breastfeeding in the USA [22, 23] and food choices in Mexico [24] and successfully increased knowledge and improved social support and family nutrition in Kenya [25].

Berkman and Krishna (2014) presented a conceptual model that links social contexts and networks to health outcomes. They embed social networks into the larger cultural and socioeconomic context in which exist these relationships of interaction between people. Social relationships influence people through various psychosocial mechanisms that affect the behaviour or health of an person [26]. These psychosocial mechanisms are social support, social influence, social engagement, access to resources and negative social interactions. Social support describes the support received from others in one’s social circle and is categorized as instrumental, informational, emotional and appraisal support [26, 27]. It is the most studied of the psychosocial mechanisms [26]. Social influence can be any attempt to modify behavior, and is described as peer pressure and social comparison guided by social norms [26]. Social engagement, or network participation, defines social roles and provides companionship [26]. Access to resources through social networks can influence for example access to healthcare or job opportunities [26]. Lastly, negative social interactions can include abuse, demands or criticism [26]. Though the model is epidemiological by nature, the psychosocial mechanisms may be useful in structuralizing factors that make up the social realities wherein IYCF occurs.

Previous studies found social support offered to mothers by spouses to increase breastfeeding duration, and support offered by female family members to increase the duration of both breastfeeding and exclusive breastfeeding [28]. Exclusive breastfeeding support was linked to increasing the likelihood of exclusive breastfeeding through increasing maternal self-efficacy [29]. When support was provided by fathers [30, 31] and grandmothers [31], social support also increased proper complementary feeding, as measured by meal frequency, dietary diversity [30–32] and minimum acceptable diet [32].

While all of the psychosocial mechanisms influence behaviour in determining access to resources, opportunities and information, and constraints on behaviour [26], the mechanisms other than social support have received little attention in the African context. Consequently, this study explores whether the different psychosocial mechanisms of interaction between people manifest themselves in IYCF in remote villages in eastern Uganda and if these could be used to describe the social realities surrounding IYCF.

## 2. Materials and methods

### 2.1. Study area

The study was conducted in the Kirewa subcounty of the Tororo district, in Bukedi, Uganda. According to the Ugandan Demographic and Health Survey (2018), poverty is prevalent in Uganda, especially in rural areas. Kirewa is one of the poorer areas of the country, in which farming is the main livelihood and low education and literacy levels exist. Ugandan women marry at a young age (in Bukedi at 17.8 years; national median 18.7 years) and polygamy is common. Women in the Bukedi region give birth to 6.1 children on average (national mean 5.4), with 22% of teenagers becoming mothers. [33]

The under-5 mortality rate in Uganda decreased from 151 deaths per 1000 live-births in year 2000 to 64 deaths per 1000 live-births in 2016, and 70% of babies were born in hospitals. Of all babies born, 66% were exclusively breastfed and 15% of 6- to 23- month-olds received a minimum acceptable diet. Of children aged 6–59 months, 29% were stunted and 11% underweight. Approximately 50% of all children and 32% of women were anaemic. [33]

Health centers may be located far away from family homes and healthcare workers are few in number; therefore, community health workers, called village health teamers (VHT), are important first contacts to health services for families. Selected from local communities, VHTs are volunteers who visit families to educate and refer patients to the health facilities. [34] The VHT program was established in Uganda in 2001 [35].

### 2.2. Data collection and analysis

The study participants were recruited from community members by a local midwife between October 7–10, 2018, immediately followed data collection. The data collection aimed to explore infant feeding perceptions, practices, and determinants, including social aspects. See the supplementary material for the semi-structured focus group discussion (FGD) (S1 Fig) and interview (S2 Fig) guides.

We conducted two FGDs with mothers, two FGDs with fathers and two FGDs with grandparents. The FGDs aimed to yield information on the thoughts and opinions of different family members whose actions directly affect IYCF. The themes of the FGDs were: care-givers´ opinions about the needs for good growth of a child, the roles of various care-givers in taking care of children, and support from family-members and others. Additionally, we conducted interviews with two clan leaders, six VHTs, four health care workers (HCWs) and a traditional healer. These interviews aimed to give a broader view of IYCF in the area, information on the opinions and attitudes of these figures of authority, and the relationship between them and the families. All these different groups of informants were included to ensure data quality through data triangulation and get a wide scope of the social realities surrounding IYCF [36]. The participants for the FGDs and interviewees were purposely selected by a local midwife. We asked her to recruit 8 participants in each category: mothers, fathers, grandmothers, and grandfathers. The inclusion criteria for the parents were that they should have at least one child aged 5 or younger, and for the grandparents that they should have at least one grandchild of any age, and that they lived in the study villages and were able to attend on the date of the FGDs. One father with a six-year-old child was included as not enough participants were found in a reasonable timeframe. All recruited parents and grandparents were not able to join, and thus seven mothers, six fathers, six grandmothers and five grandfathers participated in the FGDs. The main reason for refusal was not being able to leave work and family duties to attend the FGDs. All healthcare workers except the two who acted as interpreters were included in the interviews, as were both clan leaders and the six VHTs from the study area. Moderation of discussions in the FGDs involved a female researcher (KK) trained in qualitative data collection, together with a male or female interpreter, both trained healthcare workers from the study community. To achieve consistency, the individual interviews were conducted by the same female researcher (KK) and the same interpreters. The interviewer asked participants questions in English and the interpreter asked these in Jopadhola and gave their answers in English, which were transcribed from audio recordings.

All participants provided their written informed consent. The Makerere University School of Health Sciences Research and Ethics Committee provided ethical approval, Approval Number 2018–026.

During data analysis, we deductively searched the data for any indications of elements that could influence child feeding and health using the psychosocial mechanisms introduced by Berkman and Krishna [26] as the broader themes. These themes were social support, social influence, social engagement, access to resources and negative social interactions [26]. The last mechanism, the influence of person-to-person contact in disease spread, is not consider here due to irrelevance. Initial codes were created under these themes according to existing literature. However, as it was unknown how the themes would be manifest in the data, inductiveness in the coding was allowed to catch all meanings of the data and thus codes were added and modified in the process. Everything in the interviews and FGDs relevant to ICYF and child health was included in the selected data. After reading the transcripts multiple times, the lead author (LS) identified segments of text with similar meanings and labelled them with codes. Subsequently, the codes were carefully evaluated in collaboration with the researcher (KK) who had collected the data to ensure coherence with the field experience, further assuring quality of the data. Sub-categories were then created under the themes, under which the final codes fell. The coding matrix showing the themes, sub-categories and codes is available as S1 Table.

## 3. Results

### 3.1 Participant characteristics

The mothers in the study were aged between 22 and 30 years and had between two and five children. Over 50% of the mothers had gone to primary school, and approximately 33% had attended lower secondary school. The fathers were somewhat older than the mothers, as approximately 67% were 40 years or older. The fathers also had more children than the mothers and were better educated, as 66% of the fathers had attended lower secondary school. Most grandmothers in the study were aged in their 40s and had on average four children and between one and six grandchildren. The grandfathers in the study were aged in their 50s or 60s and had on average five children and five grandchildren. All grandfathers had attended either primary or secondary school. Peasant farming was the most common occupation. The village health teamers and healthcare workers were heterogenous in their age and number of children and were somewhat better educated than the other study participants. These are presented in Table 1.

**Table 1:** Participant characteristics.

	Mothers,	Fathers,	Grand-mothers,	Grand-fathers,	VHT,	HCW,	Clan leaders,
	n (%)	n (%)	n (%)	n (%)	n (%)	n (%)	n (%)
Interviewees	7	6	6	5	6	4	2
Age, years							
19–29	6 (86)	1 (17)			1 (17)	1 (25)	
30–39	1 (14)	1 (17)			1 (17)	2 (50)	
40–49		4 (67)	5 (83)		1 (17)	1 (25)	
50–59			1 (17)	3 (60)	2 (33)		1 (50)
60–69				2 (40)	1 (17)		
>69							1 (50)
Child count. average [range]	3.6 [2,5]	7 [5,10]	3.7 [1,5]	5 [4,7]	5.3 [2,9]	2.8 [1,6]	6.5 [5,6]
Age of youngest child. years							
0–2	6 (86)	3 (50)			3 (50)	2 (50)	
3–5	1 (14)	2 (33)	1 (17)		1 (17)		
Over 5		1 (17)	5 (83)	5 (100)	2 (33)	2 (50)	2 (100)
Grandchildren. average [range]			2.3 [1,6]	4.5 [2,14]	2.3 [1.4]		10 [2,28]
Education							
Less than primary school	3 (43)		1 (17)				
Primary school	2 (29)	2 (33)	3 (50)	3 (60)	1 (17)		2 (100)
Lower secondary school	2 (29)	4 (67)	1 (17)	1 (20)	5 (83)	3 (75)	
Higher secondary school				1 (20)			
College				1 (20)		1 (25)	
Occupation							
Farmer	5(71)	6 (100)	5 (83)	3 (60)	6 (100)		1 (50)
Seamstress	1 (14)						
Hairdresser	1 (14)						
Teacher			1 (17)	1 (20)			1 (50)
Civil servant				1 (20)			
Nurse						1 (20)	
Midwife						2 (40)	
Health officer						1 (20)	
Traditional healer						1 (20)	

VHT = village health teamer, HCW = health care worker

### 3.2. Manifestations of psychosocial mechanisms relevant to infant and young child feeding

**Fig 1:**
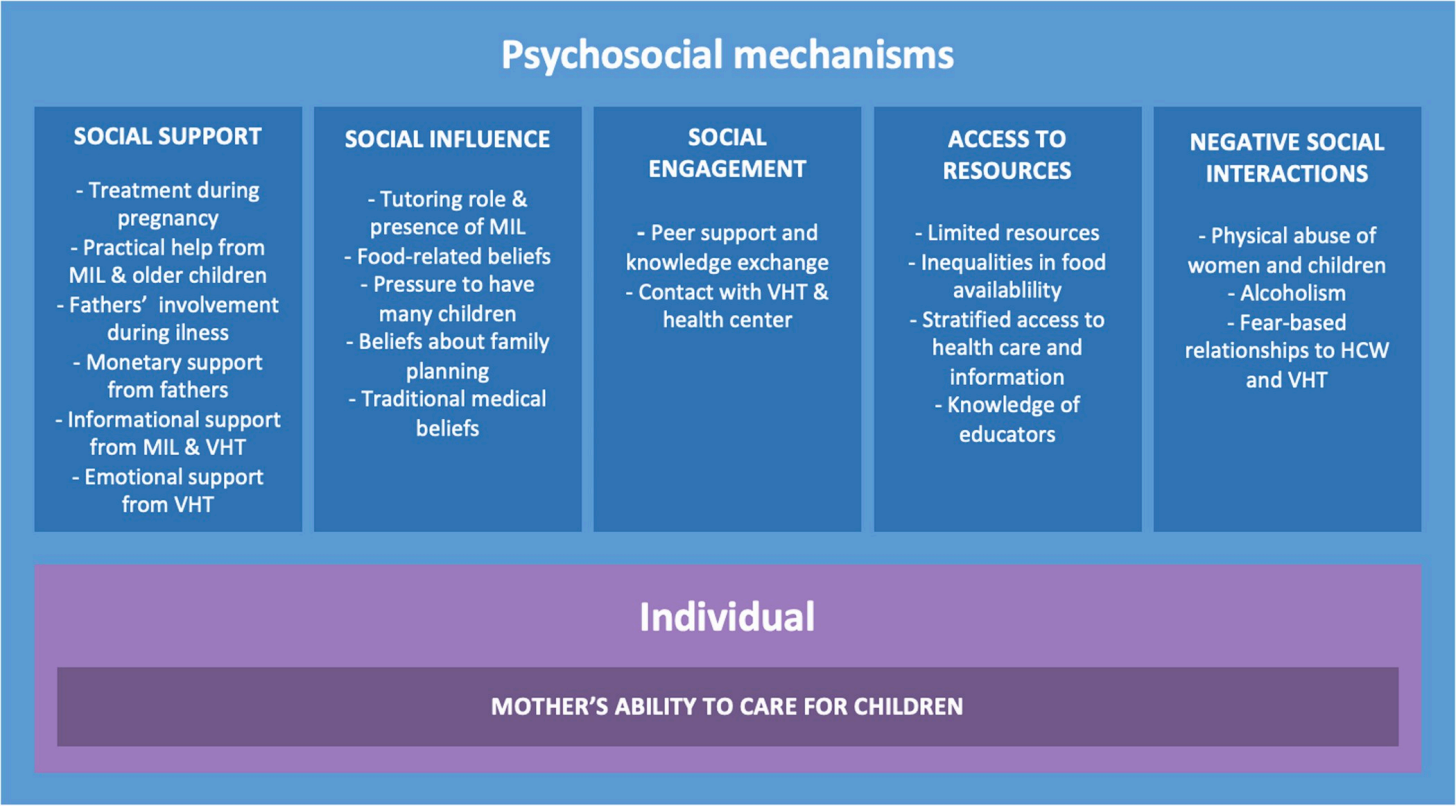
Manifested psychosocial mechanisms that may affect mothers’ ability to properly feed children. MIL = mother-in-law.

Fig 1 shows how different psychosocial mechanisms related to IYCF were manifested in the data.

#### 3.2.1 Social support

Social support presented itself as instrumental support from grandmothers, fathers, and older children. Informational support was provided by MIL, VHT and HCW; VHTs also provided instrumental, emotional and appraisal support to mothers.

Grandmothers, usually MILs, provided practical and informational help on childcare. When a young woman became pregnant, she received much help from her husband and MIL, and her workload was drastically reduced. *‘The husband will do [her] work’* (Father #3). She was often provided with better food and new clothes. ‘*When she becomes pregnant*, *we are very happy*. *We can even buy that lady milk’* (Grandfather #4). MILs usually accompanied mothers to the hospital for childbirth. Grandmothers were described as *‘loving the children so much’* that they would happily take care of them while the mother was away. Older children aged 11 to 12 years were also expected to help with household chores and childcare. If the mother was away for a longer period, the father and children would be fed by sisters-in-law. ‘*They all bring their food and sit together’* (Father #5).

Practical help was not available to all mothers; mothers commonly had to *‘leave the hospital only hours after delivering to take care of children and duties at home’* (Midwife #2, female). Practical help with childcare was declined by alcoholic grandmothers: ‘*Those who drink alcohol don’t want to take care of the children’* (Mother #7).

Fathers did not consider it their duty to help with childcare other than by providing monetary support. *‘The fathers maybe care for animals*, *or even just to go out and chat with their friends’* (Father #2). When children were ill, however, fathers tended to become more involved. ‘*I will have to carry that child to the hospital’* (Father #2). Fathers provided money for travel expenses to the hospital and for buying additional food for the child who was ill.

Mothers made decisions about the food to feed their children; however, mothers were reliant on the fathers’ provision of food items. This arrangement was described as a cause of child malnutrition in the area because fathers did not know what foods were needed by the children: ‘*It is only the mothers who come for health education’* (Midwife #2, female). Thus, the VHTs strived to deliver IYCF messages to both parents whenever possible: ‘*After teaching him and leading him to think it was his idea*, *the father will offer an egg from his chicken to the child’* (VHT #4, female).

VHTs helped mothers overcome fears that prevented them from attending health services: ‘*I will become their friend*. *Then I encourage her*: *“I am going to escort you to the health facility*.*” Then she accepts and we go’* (VHT #2, female). VHTs also helped mothers process HIV diagnoses and were valuable mediators between the husband and wife: *‘The couples that have misunderstandings come together with the VHT as their mediator’* (Midwife #1, female).

#### 3.2.2. Social influence

Social influence on childcare and IYCF manifested mainly through MILs, who were constantly present and taught mothers how to run the home and take care of children: *‘The mother is supposed to tell the wife what to feed to the children’* (Father #7). A MIL was the primary go-to-person when a young mother needed help, and her insight and experience was valued. However, both grandparents primarily decided if a child was sick enough to need hospitalization. ‘*They will check that child´s health*. *Then they will decide whether to go to the hospital’* (Mother, #3).

Grandparents maintained old traditions and beliefs through which they influenced IYCF. It was believed that pregnant women should not breastfeed, and that babies needed other foods beside breastmilk: *‘Grandparents advise to initiate food when the baby is three months’* (Mother #2). Several food-related beliefs influenced the type of food fed to children, who were believed to only need porridge and staples such as cassava. The list of unsuitable complementary foods included readily available nutritious foods such as dried fish. Although traditions were valued in the area, most parents and grandparents were open to education from the HCW. *‘If there are any challenges with this child*, *we nowadays send them to the health facility*. *From there we get a better outcome’* (Grandfather #4).

Social influence, led by village elders, favoured large families. The grandfathers, fathers and clan leaders talked about reproducing as the purpose for their existence: *‘We are here because of producing’* (Clan leader #1). Having many children was considered to bring status, security and joy and it was the measure of a woman’s worth: *‘He can give every support that is needed and can also increase his love to her’* (Father #1). Polygamy was common in the area [33] and men, whose parents were dissatisfied with their son’s number of children, were pressured to take another wife: ‘*I advise them to add and if it is not possible to add*, *you get another woman*!*’* (Grandfather #5). Some interviewees, however, discussed how many children could become a burden that fathers had not anticipated, resulting in fathers spending excessive time ‘boozing’ or even leaving their families.

Contraceptives were used in the area [33]; however, their usage was often hidden from husbands, and some mothers were afraid of family planning due to incorrect beliefs: ‘*There are myths like*, *you don’t receive your periods and your stomach grows*, *eh*?*’* (Midwife #2, female). Mothers who used family planning services were viewed as selfish and lazy.

#### 3.2.3. Social engagement

Social engagement was not discussed beyond casual contact with family and friends and attending church, although VHT visits added to the social encounters of mothers.

Mothers formed groups with friends and sisters-in-law; in these groups, mothers could discuss childcare and other matters. *‘They share their problems with the sisters-in-law’* (Grandfather #1). Other extra-household social engagements were not much discussed; however, it was common to attend church and some mothers reported being in contact with their childhood family.

Young married women were not allowed to interact with their old friends, and many lived far from their childhood families. ‘*When you are married*, *you have to sit in one place’* (Grandmother #1).

#### 3.2.4. Access to recourses

Access to resources was manifested as inequalities in access to food and healthcare and, therefore, health and nutrition education.

As recourses were scarce, families had to prioritize their expenditures. Education in the area was low [33] although highly valued. Consequently, school fees were often prioritized over food purchases and food grown was sold to pay for school fees: ‘*We can sell food to pay school fees’* (Mother #7). The lack of land for cultivation coupled with large families was described as the major reason for malnutrition in the area: ‘*You have to divide*, *then you don’t have enough food’* (Midwife #1, female).

Women and children usually ate their meals separate from men: *‘Men sit at the table*. *The children and their mothers*, *they sit on the ground’* (Grandfather #4). Available food was not equally shared between family members. The most nutritious foods were status symbols and were, out of respect, served to men. For example, a chicken was usually kept in reserve for visitors: ‘*She cooks for them well and slaughters a chicken’* (Grandfather #2).

With restricted resources at the health centre, patients with the most severe conditions were prioritized. Likewise, VHTs were short staffed and although they were meant to visit all families, even they reported prioritizing families with, for example, severely malnourished children: ‘*Mostly we go to those families that are malnourished where we emphasize a lot’* (VHT #5, female). Poor, HIV infected families that had gotten the most education were recognized as the healthiest in the area: ‘*They constantly get education on how to take care of their families because they frequently go to the health facility*. *Their children are even the healthiest’* (VHT #2, female). With poverty being a strong determinant of malnourishment, the more educated healthcare personnel focused on educating mothers on the types of foods to buy with their existing funds: ‘*There is little food*. *But when they eat little of nutritious food*, *the body can improve*. *When eating much of not nutritious food*, *the body cannot improve’* (Midwife #1, female).

However, not all HCWs were highly educated and the VHTs lacked knowledge of IYCF. ‘*I want to get more education on nutrition of the children’* (VHT #4, female). For example, stunting was not viewed as a malnutritional condition, rather a genetic trait or a condition caused by evil spirits. ‘*I don’t normally talk about that because it could be a gene that they are short’* (VHT #4, female). The health educators’ lack of knowledge restricted the mothers’ access to correct health information and mothers were further confused by traditional healers and beliefs. A traditional healer explained how he could even heal stunting: ‘*I mix the herbs*. *I ask 50 000 shillings from this family and one goat*. *After this the child gets well’* (Traditional healer, male).

#### 3.2.5. Negative social interactions

Negative social interactions included fear and physical abuse that were culturally related to respecting those higher in the family hierarchy.

‘*Good behaviour*’ and respect were highly valued, and both women and children were physically abused for misbehaviour. *‘Children who are beaten they have respect because they have fear* (Mother #1). Men were regarded as ‘*superior beings*’, whose wives should obey them. *‘I talk and you immediately act’* (Grandfather #3). Physical strength was utilized to uphold hierarchy; domestic violence or ‘*marital problems’* were abundantly discussed during the interviews. Fear was believed to be the best motivator for change; fear sometimes existed between mothers and VHTs and HCWs. Hygiene and childcare messages were often not delivered in a supportive way, but rather in a way that induced fear in the hope of forcing mothers to change their practices. ‘*If the baby is dirty*, *the VHT will say ‘You have shamed me*! *Therefore*, *I am to take you to the subcounty and so shame you’* (Mother #6). Attendance at health clinic prenatal appointments was mandatory for both parents; however, only about half of the mothers were accompanied by their husband. Reasons included HIV testing and the resulting stigma, being unable to implement recommendations and inappropriate appearance. An additional reason was the perceived unkindness of the HCWs.

Alcoholism or ‘boozing’ was the main reason mentioned for restricted support and family finances. ‘*After selling the goat*, *he will give me some money and then he will go and use the rest for drinking’* (Mother #7). Alcoholism was less common, but widespread, also among women. Clan leaders considered their duties included resolving marital issues to achieve ‘*peaceful families*’ and encouraging men not to spend all their money on alcohol. Grandparents also helped to resolve marital disputes: ‘*They will discuss what causes the violence’* (Mother #4).

## 4. Discussion

IYCF are social practices affected by multiple factors in the social context wherein a mother and child reside [8]. Consequently, we set out to explore the social realities in remote villages in eastern Uganda. Furthermore, we wanted to see whether the different mechanisms of interaction between people as described by Berkman and Krishna (2014) were manifested in IYCF and could be used to describe the social realities surrounding IYCF. We learned that social behaviours were deeply rooted in the Ugandan local culture, and expressions of the five sociopsychological mechanisms were found in the data.

Our main finding was that cultural norms and social roles created conflict between tradition and ideal IYCF. The role of HCWs, especially VHTs, was important in changing beliefs and norms; specifically crucial was the role of VHTs as mediators between the formal healthcare and community. The beginning of change was noticeable by some grandparents accepting advice from the HCWs, and foods previously available only to men becoming available to other family members.

However, no change was visible in the idolization of large families, despite the acknowledgement of problems arising from population growth and the promotion of family planning by health care professionals. Large family size was an established cultural restrictor to ideal IYCF. Since the social status of young women was tightly connected with bearing children, changing the size of their families was difficult for mothers. Village leaders opposed contraceptive use, along with grandparents and husbands, as recorded previously [37]. In Ethiopia, religious community leaders were recruited to promote safe motherhood [10]; husbands’ support was positively associated with contraceptive usage in Ghana [38]. Child spacing is important for the nutrition and health of both women and children [39] with beliefs hindering, for example, the breastfeeding of older babies during new pregnancies [40].

Mothers prominently influenced IYCF, although they were subject to their MIL in important childcaring decisions. Thus, MILs were highly influential in IYCF practices. MILs usually accompanied mothers to the hospital for birthing. Having someone accompany mothers to the health facility reportedly increased the likelihood of facility delivery in Ethiopia [41] and Kenya [42]. Likewise, receiving social support during pregnancy and childbirth increased the likelihood of facility delivery [41]. MILs also provided young mothers with practical help, which is important for IYCF outcomes [9, 29, 43] as maternal workloads have reportedly been a major constraint on child feeding practices in Uganda [11, 14]. Despite being generally supportive of breastfeeding, grandmothers rarely support exclusive breastfeeding [16, 44, 45], as seen in our study. Breastfeeding support has helped to decrease beliefs of insufficient breastmilk [46, 47], which is a common reason to stop exclusive breastfeeding [14, 37, 40]. MILs also advised on suitable complementary foods, advice that was influenced by beliefs about unsuitable foods and digestive ability. These beliefs are common in Africa and often result in the exclusion of nutritious foods, such as animal products, from children’s diets [14, 47, 48]. Therefore, educational pursuits should focus on addressing incorrect beliefs and stress the importance of animal source foods and vegetables to small children, in addition to teaching women how to prepare such for safe feeding to infants. Considering their influence on IYCF, including MILs in IYCF education would be important.

The study society was highly patriarchal, and fathers were minimally involved in practical childcare. In contrast, fathers in Tanzania reported enjoying being more involved in IYCF after being prompted to do so in an intervention [49]. The provision of monetary instrumental support was experienced as the father’s primary contribution, as elsewhere in Uganda and in Ethiopia [10]. Additionally, patriarchal society influenced the types of foods available to women and children, as elsewhere [40]. Beliefs about unsuitable foods for children, coupled with the restrictions in food availability by the patriarchal culture, led to very restricted IYC diets [47, 48]. However, educating fathers increased IYCF support received by mothers [25], as was seen in our study. Furthermore, Ugandan fathers with more nutrition education than their spouses took responsibility for providing their children with proper foods [11, 50], and fathers’ support of complementary feeding was associated with better diets in Nigeria [30]. In addition, fathers’ support strengthened the association between maternal decision making and good complementary feeding practices [30]. Fathers were acceptant towards IYCF education when such was provided during their natural gatherings outside the home [51].

The patriarchal hierarchy was upheld by asserting control over others through physical abuse. Spousal violence was experienced by 56% of Ugandan women, and 85% of children were violently disciplined [33]. While not constant, domestic violence was associated with poor IYCF practices [52–54]. Domestic violence was accelerated by alcoholism [54], with 84% of women whose husband got drunk often experiencing domestic violence [33]. Alcoholism has been described as a coping strategy for mental health problems induced by poverty [55], while also further reducing the resources available for child feeding and support [33, 54]. The culture of fear-induced control was also seen in mothers’ relationships with HCWs and was described as affecting health care attendance [56]. Men’s willingness to accompany their wives to prenatal appointments was mainly affected by their unwillingness to attend HIV testing, as also reported elsewhere [57]. In contrast, men living in urban Uganda described their willingness to support their wives by attending their appointments and participating in the delivery. Nonetheless, fathers felt ignored and confused about being encouraged to attend, as there was physically no room for them in the delivery room [58].

Poverty was a large reason for restricted resources in the area, as in many parts of Uganda [33], as well as increasing stress and restricting well-being [55]. A recent World Bank (WB) report found that poverty in Uganda had not decreased since 2013, and called for improved education and increased income-generating opportunities among the rural poor and the implementation of social protection [59]. As expressed in our data, school fees competed with food purchases, stressing the importance of free education. Uganda has yet to succeed with its pursuit to offer free education [60], while Kenya demonstrates how substantial investments in the educational sector have resulted in increased school enrolment and improved school performance [61]. Despite of this, even Kenya faces difficulties in providing equal educational opportunities for the rural poor [61]. Non-farming income generating activities in rural areas are considered crucial for poverty reduction in developing countries also other than Uganda [62]. Projects have aimed to do so by empowering women through involving them in income generating activities [63]. A study in Uganda found that such an approach led to marital problems and women being disrespected in the traditionally patriarchal community and recommended a whole-family approach to be implemented instead. In addition, women are often already overburdened and their sole participation in income generating activities in the area were insufficient to lift families out of poverty, while targeting both men and women might potentially be more successful. [63] Our results support the idea of a whole-family approach in rural development and education.

Poverty and seasonal food insecurity leads to monotonous and insufficient diets [54, 64], as in our study area. Notwithstanding the prevailing food insecurity and restrictions in children’s diets, VHTs reported great improvements in child nutrition and health stemming from education. Access to health information can improve IYCF practices [65] and child anthropometrics, even in food insecure areas, which has also been recorded elsewhere [4]. Caregivers’ low knowledge on breastfeeding and complementary feeding were previously listed as the main barriers to ideal IYCF in Uganda, together with cultural practices [14]. However, access to health information was not equal for all families, as those with illnesses and obvious malnutrition were prioritized. Many African countries, Uganda included, suffer from a severe shortage of HCWs caused by an array of difficulties in the healthcare sector including inadequate training capacity, weak governance, insufficient resources of the public health sector, rapid population growth and international migration of trained HCWs [66]. The role of CHWs in nutrition, hygiene, and health education has consequently been emphasized [67, 68] especially in remote locations, where CHWs are often the only information source in addition to family and friends [69]. Despite the usefulness of CHWs programs, CHWs often have insufficient IYCF knowledge [68, 70], affecting their ability to provide correct IYCF information, which has been linked to poor IYCF practices [71, 72] and even child stunting [73]. However, VHTs in the study area were reported to gain confidence and teach more convincingly when provided with teaching tools in the form of educational videos in mobile phones [51]. Such mobile solutions may have great potential in equalizing access to health knowledge, as the WB outlines a digital transformation as a key driver towards growth and poverty reduction [59].

VHTs gave mothers social support that was not available from other sources. Young mothers had restricted social circles; the limited nature of outside influence increased the importance of the role of the VHT visits, as the availability of multiple social networks was linked to greater health knowledge [74]. Also literate community members and a regular market were linked to health knowledge [74], suggesting the importance of social engagement to spreading IYCF knowledge. In addition to information sharing, groups of neighbours in Kenya traded food to diversify diets [25]. In cultures where young married women’s social engagement is restricted, finding socially accepted ways to increase such, for example through women’s groups [75, 76], or integrating health education to church activities [77], may be good ways to increase exposure to IYCF and health information. CHWs are another culturally accepted social contact, though their relationship with families, and thus their ability to offer social support, is sometimes lacking [78, 79]. We previously found that relationships between CHWs and mothers could be improved by increasing the credibility of CHWs and shifting their position in the community [51]. Furthermore, CHW home visits were associated with increased antenatal care (ANC) attendance also in Mali [80]. However, no significant effect on ANC attendance was noted in hard-to-reach parts of Uganda [81] or Tanzania [82]. In India and Ethiopia, CHWs accompanied their clients to the health facility for delivery [10, 83]; in Ethiopia, CHWs reported assisting mothers after delivery with cooking and other tasks [10].

### Strengths and limitations

Studying social realities in a remote location is valuable, as cultural traditions and norms prevail. Through the model by Berkman and Krishna (2014), and by including multiple different informants who all influence IYCF, we were able to get a comprehensive description of the social realities influencing IYCF. In addition to this, quality of data was strengthened through using the same interviewer/moderator for all interviews and FGDs, her keeping field notes and transcribing the data soon after the interviews. Moreover, the data was primarily analyzed by a researcher who was not present during the interviews, ensuring objectivity, while the meanings and coding were agreed upon the two researchers. [84]

A limitation concerns the purposeful selection of the participants by a local midwife, as such selection may have been based on participants’ proximity to the health centre. Also, the average age of participants in our father groups was higher than the mothers; therefore, opinions of the younger generation may have been unexploited. Ours was a small sample at one point in time and further studies are needed. However, the framework employed proved useful for studying the social relationships affecting IYCF. Finally, the presence of western interviewers could have influenced the answers, and the interpreter may have left out details as his answers were not as long as the interviewees.

## 5. Conclusions

A comprehensive understanding of the social realities surrounding a mother-child pair is needed when aiming to influence IYCF through for example educational interventions. The different psychosocial mechanisms were well suited for creating a framework to describe such social realities in rural Uganda.

Through these, the following restrictions to proper IYCF emerged: First, attitudes favouring large families, coupled with poverty and patriarchy, limited the availability of foods for IYCF. Second, fathers as providers and decision makers were not involved in IYCF. Thus, future education should target attitudes towards family planning and the knowledge of all family members’ on IYCF. IYCF education should focus especially on eradicating false beliefs and making nutritious foods culturally available to all family members. It was also clear that educating merely mothers is insufficient in cultures where social influences on IYCF are strong. Further studies are needed on how such education should be provided, as it may be culturally appropriate to educate men and women in their own groups.

In addition, strengthening the position of VHTs as family advisers should be prioritized. Coupled with proper educational tools, VHT support could improve IYCF in the remote areas of Uganda.

While our explorative study found manifestations of the psychosocial mechanisms in conjunction to IYCF, the complete model, including social network analysis, should be utilized in further studies of social impacts on IYCF. Furthermore, the attitudes of the younger generation should be studied for their IYCF related social perceptions.

## Supporting information

S1 Checklist (DOCX)

S1 Fig The focus group discussion guide for the caregivers of young children in Kirewa subcounty. (TIF)

S2 Fig The interview guide for the individual interviews of healthcare workers, village health teamers, and clan leaders in Kirewa subcounty. (TIF)

S1 Table The coding matrix for the data. (PDF)
