# Supplementary figures and images for: Social realities in remote villages: Infant and young child feeding in Kirewa, Uganda
Source: PLOS Glob Public Health. 2024 Sep 10;4(9):e0003016. doi: 10.1371/journal.pgph.0003016 (PMC11386423; doi:10.1371/journal.pgph.0003016)

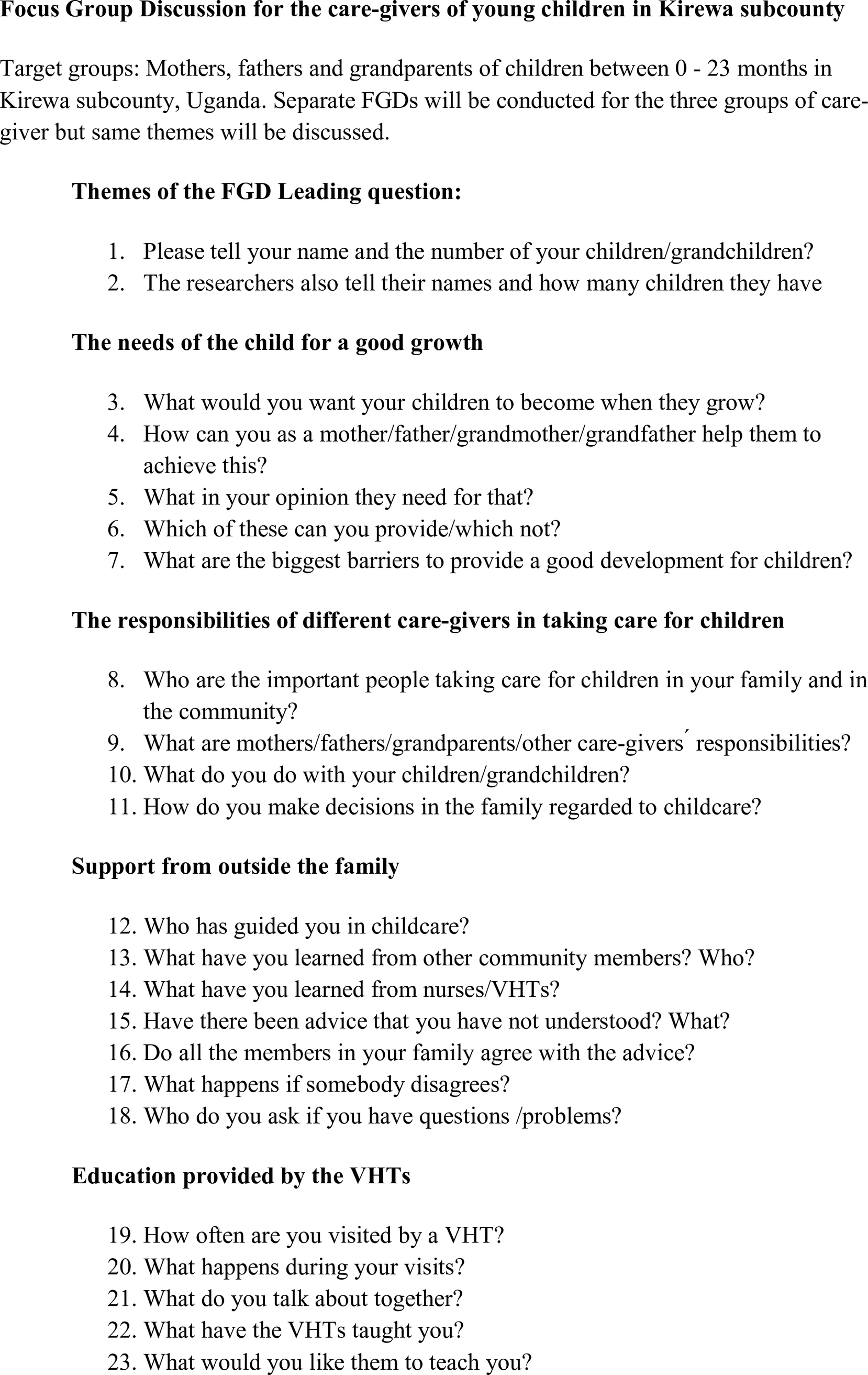

Supplement: S1 Fig — (TIF) [file pgph.0003016.s002.tif]

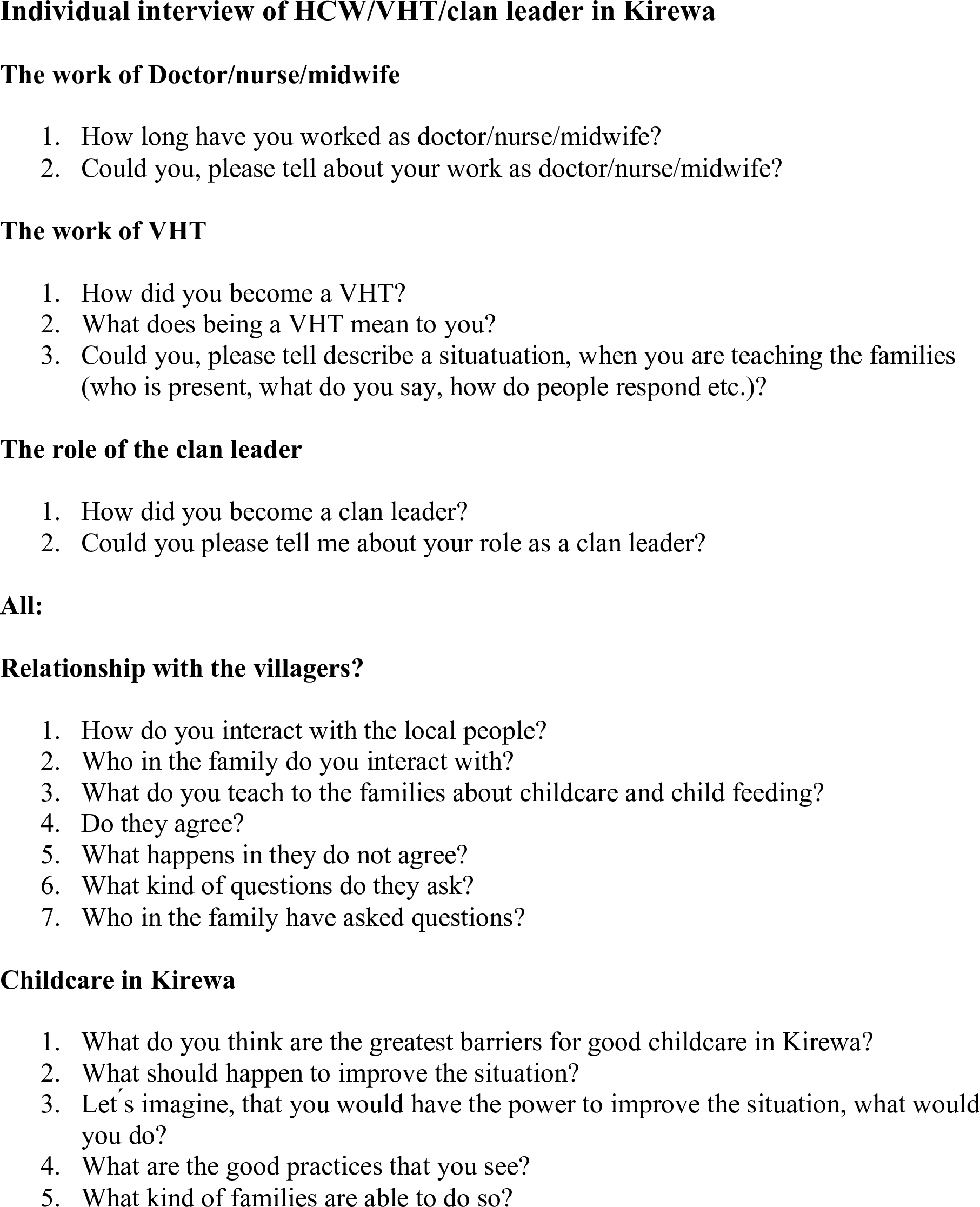

Supplement: S2 Fig — (TIF) [file pgph.0003016.s003.tif]
