# Supplementary material for: Social realities in remote villages: Infant and young child feeding in Kirewa, Uganda
Source: PLOS Glob Public Health. 2024 Sep 10;4(9):e0003016. doi: 10.1371/journal.pgph.0003016 (PMC11386423; doi:10.1371/journal.pgph.0003016)
Supplement: S1 Table — (PDF) [file pgph.0003016.s004.pdf]

Supplementary table 1. The coding matrix for the data.

themes	sub-categories	codes
social support	practical help	help from MIL<br>help from father<br>help from others
	monetary support	food purchases<br>healthcare expenses
	emotional and appraisal support	support from VHT
social influence	IYCF traditions	information from MIL<br>IYCF beliefs<br>information from HCWs
	other relevant traditions	family size<br>family planning
social engagement	contacts with family members	contact with husband's family<br>contact with childhood family
	contacts outside the family	contacts with friends<br>church attendance<br>VHT visits
access to resources	access to food	food availability<br>recourse prisonization<br>cultural access to food
	information availability	availability of HCW and VHTs<br>competence of HCWs and VHTs<br>traditional healthcare
negative social interactions	hierarchy	patriarchy<br>fear of HCWs & VHTs
	alcoholism	alcoholism among men<br>alcoholism among women

MIL = mother-in-law, VHT = village health teamer (equivalent to community health worker), HCW = health care worker, IYCF = infant and young child feeding
